# Origin of Sex-Biased Mental Disorders: An Evolutionary Perspective

**DOI:** 10.1007/s00239-021-09999-9

**Published:** 2021-02-25

**Authors:** Rama S. Singh, Karun K. Singh, Shiva M. Singh

**Affiliations:** 1 Department of Biology, McMaster University, Hamilton, Canada; 2 Stem Cell and Cancer Research Institute, McMaster University, Hamilton, Canada; 3 Krembil Research Institute, University Health Network, Toronto, Canada; 4 Department of Biology, University of Western Ontario, London, Canada

**Keywords:** Sexual selection, Sex-biased diseases, Mental disorders, Male-driven mutation, Autism, Female immunity

## Abstract

Sexual dimorphism or sex bias in diseases and mental disorders have two biological causes: sexual selection and sex hormones. We review the role of sexual selection theory and bring together decades of molecular studies on the variation and evolution of sex-biased genes and provide a theoretical basis for the causes of sex bias in disease and health. We present a *Sexual Selection-Sex Hormone* theory and show that male-driven evolution, including sexual selection, leads to: (1) increased male vulnerability due to negative pleiotropic effects associated with male-driven sexual selection and evolution; (2) increased rates of male-driven mutations and epimutations in response to early fitness gains and at the cost of late fitness; and (3) enhanced female immunity due to antagonistic responses to mutations that are beneficial to males but harmful to females, reducing female vulnerability to diseases and increasing the thresholds for disorders such as autism. Female-driven evolution, such as reproduction-related fluctuation in female sex hormones in association with stress and social condition, has been shown to be associated with increased risk of certain mental disorders such as major depression disorder in women. Bodies have history, cells have memories. An evolutionary framework, such as the *Sexual Selection–Sex Hormone* theory, provides a historical perspective for understanding *how* the differences in the sex-biased diseases and mental disorders have evolved over time. It has the potential to direct the development of novel preventive and treatment strategies.

## Introduction

Why does the prevalence of certain diseases differ in men (boys) and women (girls)? Being male is the single largest risk factor for early mortality in developed countries (Kruger and Nesse 2004). A 2008 World Health Organization survey investigating 36 diseases showed that, with a few exceptions (such as Alzheimer’s disease and other dementias and iron deficiency anemia), men had higher mortality rates than women in almost all cases of non-sex-specific diseases. On reflection, differences in the prevalence and manifestation of various diseases between sexes are not surprising considering the differences between the sexes in terms of their sex chromosomes, development, anatomy, physiology, reproductive biology, and lifestyle.

Men and women differ in terms of presentation of sex-specific diseases, including prostate and testicular cancers for men and breast, cervical and ovarian cancers for women. However, the prevalence of some common diseases that are *non–sex-specific* also differs between men and women. Men generally show a higher prevalence of cardiovascular diseases (Albrektsen et al. 2016), whereas women show a higher prevalence of certain types of arthritis and rheumatic pains (van Vollenhoven 2009). A similar pattern is present for mental disorders, with a higher prevalence of anxiety and depression in women and a higher prevalence of antisocial disorders in men (Albert 2015). The causes of mental disorders are complex and remain unclear, although family, twin and population studies have indicated that both genetic and environmental factors are likely to play important roles in their development (Chokroborty et al. 2014). Such overarching and generalized explanations do not explain the known male bias associated with several mental disorders. Further, an insight into the sex-specific occurrence of these disorders may offer novel perspectives on their underlying causes and mechanisms, revealing potential avenues for understanding mechanisms and strategies for prevention and treatment. In this appraisal, we address the issue of sex-biased mental disorders using the example of autism, a common sex-biased mental disorder affecting children (Miles and Hillman 2000; Chakrabarti and Fombonne 2001) and present an evolutionary theory to explain sex-biased diseases and mental disorders in general.

### Theorizing Autism: What do we Know?

Several theories have been offered to explain the male bias of autism. The “extreme male brain” theory proposes that the autistic brain represents an extreme form of the “typical male” brain profile (Baron-Cohen 2002). The “empathizing–systemizing discrepancy” cognitive domain metric shows sex differences: females are “empathizing,” males are “systemizing”, and autistic males and females are extremely “systemizing” (Baron-Cohen 2002). While excessive fetal testosterone was once thought to be a causal factor in autism, recent data implicate a multitude of steroid hormones (Baron-Cohen et al. 2020). According to this theory, one reason for sex differences may be that male autistic children are more likely to be diagnosed due to their extreme phenotypes.

Because females exhibit reduced autism risk, a genetic protective model has also been proposed which causes the diagnostic threshold to be higher for females than males but does not explain how or why (Lai et al. 2011; Robinson et al. 2013; Jacquemont et al. 2014; Gockley et al. 2015; Lai, Baron-Cohen et al. 2015a, b; Lai, Lombardo et al. 2015a, b; Werling 2016). Another theory proposes that sex differences in autism are associated with sex differences in grey and white matter (Torres et al. 2013; Gockley et al. 2015; Halladay et al. 2015). Substantial evidence suggests that males are more vulnerable than females beginning at conception, referred to as the fragile male hypothesis (Kraemer 2000). These theories confuse *proximal* and *ultimate* causes, i.e., causes that are immediate and of functional nature vs. causes that are evolutionary, respectively (Mayr 1961). These theories do not explain sexual disparities but instead offer potential *proximal* pathological causes for the high or low incidence of a disorder within a given sex. Therefore, it is logical to argue that the biological bases of sex differences in the brain are associated with development and involve differential gene expression patterns that cause the male brain to function and respond differently than the female brain (Trabzuni et al. 2013). More importantly, these sex differences are thought to result from evolutionary, *ultimate* causes. Evidence suggests that both genetic and epigenetic processes are inherently different for males and females and not only determine sex but also direct sexual differentiation, sexual selection, sexually antagonistic evolution and sexual dimorphism in both disease and health (Morrow 2015).

After summarizing a set of evolutionary principles with bearing on sex and gender as well as health and disease, we provide an overarching two-component theory involving sexual selection and sex hormones (SS-SH theory) to explain sex-biased differences in the prevalence of diseases and mental disorders. Using autism as an example, we show that, although the molecular mechanisms underlying diseases and mental disorders may be associated with individual risk factors or developmental anomalies, sex-mediated differences in their prevalence may be associated with the evolutionary history of sexual selection, which would affect all genes—both sex and non-sex. We propose that the male-biased prevalence of non–sex-specific mental disorders such as autism is the result of male-driven evolution leading to the following: (1) increased male vulnerability due to the negative pleiotropic effects of male-driven sexual selection and evolution for early gain of fitness; (2) increased rates of male-driven mutations and epimutations (germline and somatic) due to male-driven sexual selection and resulting in early fitness gains at the cost of late fitness; and (3) increased female immunity due to the development of antagonistic responses to mutations that are beneficial to males but harmful to females, reducing female vulnerability and increasing the thresholds for diseases and mental disorders. The SS-SH theory is presented here as a significant “first component” of the variation in sex-biased diseases and mental disorders.

### The Genetic Basis of Autism: An Overview

The primary diagnostic features associated with autism include social deficits, language impairment and repetitive behaviors. These features are often associated with a variety of other abnormalities, especially neurological, including intellectual disabilities. Some authors (Constantino and Todd 2000) have suggested that most cases of autism are inherited and polygenetic, with small genetic effects contributing to the overall risk of disease manifestation. Others (Geschwind and Levitt 2007) have argued that autism exists in two forms: “complex” autism (20–30%), which is defined by the presence of dysmorphology (minor physical anomalies) and is associated with a lower male-to-female ratio; and “essential” autism, which is defined by the absence of dysmorphology. Essential autism is common in males, has a high sibling recurrence risk and is associated with a family history of autism and autism-related disorders (Miles et al. 2005; Miles 2011). This form of autism is thought to be caused by common allelic variants that segregate in families, which would indicate the disorder’s heritability and would account for families with multiple affected children (Klei et al. 2012; Gaugler et al. 2014). In fact, extensive genome-wide association studies have implicated hundreds of genes and a variety of associated genetic pathways in autism (Woodbury-Smith et al. 2018). A recent genome-wide association study has shown tandem DNA repeats, both common and rare, to be significantly associated with Autism Spectrum Disorder (Trost et al. 2020). A more practical approach has been suggested (Fernandez and Sherer 2017) to divide Autism into clinically defined such as those caused by fragile X syndrome (4–5%) and molecularly defined such as those based on genome-wide testing (20%). The underlying cause(s) of the majority of the autism cases remains unknown.

In addition to common segregating variants, autism has also been associated with a host of de novo mutations (O’Roak et al. 2011; Gilman et al. 2011; Sanders et al. 2012; De Rubeis et al. 2014; Iossifov et al. 2014) and copy number variations (Sebat et al. 2007; Marshall et al. 2008; Pinto et al. 2010, 2014). Interestingly, the overwhelming proportion of de novo mutations have been shown to be paternal in origin and are associated with paternal age (O’Roak et al. 2011). Furthermore, a significant number of de novo events have been found to be recurring, affecting neuronal gene products (O’Roak et al. 2012). The identification of de novo mutations suggests the potential involvement of major disruptive (missense or nonsense) mutations that affect brain-expressed genes (Sanders et al. 2012). Although such mutations account for only 40% of de novo mutations, they have been shown to affect high-connectivity protein genes. These results suggest that recurring protein-altering mutations in highly connected brain-expressed genes may represent candidate causative mutations associated with at least some autism cases (Neale et al. 2012; Samocha et al. 2014).

More recent research exploring the causes of autism has focussed on epigenetic mechanisms, particularly DNA methylation (Nardone et al. 2014; Lad-Acosta 2015; Loke et al. 2015, Tremblay and Jiang 2019). Epigenetic changes have the potential to affect gene expression in response to environmental effects—including during neurodevelopment—without altering the gene sequence (Feng et al. 2007). Genes associated with the epigenetic pathway, which have been identified through a variety of approaches, constitute a sizable proportion of candidate autism genes, and are included in the autism database (http://gene.sfari.org/). Even at this relatively early stage of epigenetic research, a small number of replicated methylation differences have also been associated with autism (Ladd-Acosta et. al 2014; Nardone et al. 2014). These results support the potential contribution of epigenetic processes to the etiology of autism. The involvement of epigenetic changes, particularly DNA methylation, is also relevant in the context of sex differences. DNA methylation, which is known to differ between the two sexes, appears early, and affects imprinted genes and X-inactivation (Skuse 2005). More importantly, an individual’s sex has the potential to determine DNA methylation–based, sex-specific gene expression at any stage of development and differentiation, including during neurodevelopment. Epigenetic differences may also affect paternal sperm, which has the potential to contribute to the development of autism in offspring (Feinberg et al. 2015).

In summary, several major breakthroughs related to the genetic determinants of autism have been reported, including the identification of many common alleles (single-nucleotide variations and copy number variations) that currently segregate within the population; prezygotic and postzygotic de novo mutations (single-nucleotide variants and copy number variations); and epigenetic changes that impact gene expression (DNA methylation and histone modification). Ultimately, these determinants can cause imbalances in highly regulated neurodevelopmental processes that manifest as variations in disease prevalence and manifestation. The results of published studies argue that autism, like most mental disorders, is genetically heterogeneous, involving many genes that are implicated in a variety of pathways that affect neurodevelopment and brain function. Furthermore, these genes may be altered by a variety of mutational and epimutational mechanisms, as supported by the extensive variability in the manifestations of autism.

### The Sex-Biased Prevalence of Autism

Both the prevalence and the severity of manifestation of autism differs in boys and girls; recently, an increasing number of studies have begun to explore the low prevalence of autism in females (Lai et al. 2011; Lai et al. 2015a, b). Overall, the reported male-to-female ratio among individuals with autism ranges from 4:1 (Chakrabarti and Fombonne 2001) to 2–3:1 based on recent combined data (Lai et al. 2013; Lai et al. 2015a, b). Individuals with essential autism are more likely to be male (6.5:1) (Miles 2011), with some earlier studies reporting disparities as high as 23:1 (Hillman et al. 2000). However, a recent meta-analytical review showed a ratio of 3:1 (Loomes and Mandy 2017). Autistic girls tend to be diagnosed later than autistic boys and tend to have more severe symptoms (Shattuck et al. 2009; Giarelli et al. 2010; Begeer et al. 2013). However, the differences are not straightforwardly dictated by sex biology and are interwoven with the patient in the context of sex and gender (Lai and Szatmari 2020

The reduced incidence and increased severity of autism in females may be attributable to many factors. First, sex-limited factors may be involved, such as the sex-determining region Y (SRY) gene, X-inactivation, sex-limited genomic imprinting, Y-linked inheritance and unique X–Y interactions (Beaudet 2017). Second, the levels of gene expression in different brain regions may differ in males compared to females (psychENCODE 2015; Hu et al. 2015). In addition, female genotypes may be developmentally protective (Werling and Geschwind 2015) and may require higher thresholds for mutations or gene expression to manifest the same levels of severity (Gockley et al. 2015; Lai et al. 2015a, b; Robinson et al. 2015). Third, male fetuses may be more susceptible to developmental and environmental factors (Hu et al. 2015; Werling and Geschwind 2015). Fourth, males and females may differ in various etiological aspects; for example, female autism may represent a more complex type of autism caused by multiple factors (Geschwind and Levitt 2007). Finally, the male-biased prevalence may not be unique to autism, and sex differences in the susceptibility of normal neurodevelopmental processes to environmental perturbations, rapid neonatal brain growth and incomplete neurodevelopment may result in additive effects on disease prevalence in general (Table 1). However, one thing appears to be for sure: the higher levels of sex bias in essential autism as compared to those caused by de-novo mutations make sense as segregating genes causing essential autism would have had more time to evolve sex-biased differences.

**Table 1:** Male-driven processes producing male-biased evolutionary changes in the genome

Male-determining genes	In combination with other genes expressed later in life, SRY—the male-specific, sex-determining gene—sets the stage for lifelong hormonal effects on sexual and non-sexual traits (Gubbay et al. 1990; Sinclair et al. 1990; Raymond et al. 1998; Bourc’his and Bestor 2006; Arnold 2017)
Male-driven increased rates of mutation	Mutation rates are male-biased, with mutations occurring at higher frequencies in males than females and are higher than be explained by number of germ cell divisions (Miyata et al. 1987; Drost and Lee 1995; Ellegren and Fridolfsson 1997; Hurst and Ellegren 1998; Ellegren 2007; Keightley 2012; Lynch 2016)
Male-biased somatic mutations and epimutations	Cell type specific somatic mutations are a new addition to the measures of genetic differences ( Milholland et al. 2017) and they play a critical role in sex-specific oncogenesis (Lopes-Ramos et al. 2020). A pan-cancer analysis showed that 15% of the genes mutated in tumors had sex biased somatic mutations that are more prevalent in male tumors (Li et al. 2018). Sex biased epigenetic changes, particularly DNA methylation are known to exist across different tissues including the brain (Xu et al. 2014)
Male-driven purging of deleterious mutations	Sexual selection through male–male competition would lead to the elimination of deleterious mutations not only in sexual traits but in the whole genome (Whitlock and Agrawal 2009)
Sex-limited genomic imprinting	Male imprints are established early in life and persist longer, resulting in highly sexually dimorphic imprinted gene expression (Bourc’his and Bestor 2006)
Male-biased increases in gene expression ranges	Male-biased genes show larger ranges of gene expression levels in *Drosophila* (Ranz et al. 2003; Meiklejohn et al. 2003; Parisi et al. 2004; Zhang et al. 2007)
Male-biased rates of sequence evolution	Male-biased genes show higher rates of sequence evolution (Torgerson et al. 2002; Lawniczak and Begun, 2004; Zhang et al. 2004; Khaitovich et al. 2006; Pröschel et al. 2006; Haerty et al. 2007)
Male-specific transcripts, new genes, and loss and gain of function	Males generally express more genes and proteins, such as the accessory gland proteins of *Drosophila*, for male-specific functions (Wolfner et al. 1997)
Male susceptibility to gestational perturbations	Males are more sensitive than females to adverse gestational conditions and the impact can last lifetime (Navara 2014)
Male susceptibility to environmental stresses	Males are more susceptible than females to environmental variations throughout their lives (Bale 2009; Beaudet 2017)
Male brain plasticity	Males are more susceptible than females to perturbation in gene expression associated with synaptic plasticity, possibly due to gene–environment interactions (Andersen and Teicher 2008; Lai et al. 2013; Mottron et al. 2015)
Male-driven evolution of senescence	Male–male competition for early gains in reproduction may accelerate the accumulation of deleterious mutations that affect senescence (Williams 1957; Kirkwood and Rose 1991)
Mate choice and the evolution of menopause	A male preference for younger mates would lead to the accumulation of female infertility–causing genes and the evolution of menopause (Morton et al. 2013; Takahashi et al. 2017; Chan et al. 2020)
Male-driven persistence of maternal mortality	Child marriage and repeated marriage including serial monogamy exacerbate the problem of maternal mortality making it persist longer (Jagadeeshan et al. 2019

## Evolutionary Principles in Relation to Sex and Gender in Disease and Health

Sexual selection is a significant force of evolution and sexual dimorphism. The evolutionary framework presented here for the evolution of sex-biased diseases and mental disorders primarily refers to the sex biases associated with the *prevalence*, rather than the *causes*, of diseases. In this section, we summarize a set of evolutionary principles in relation to the evolution of sexual dimorphism in disease and health and follow it by laying out a comprehensive theory to explain sex-biased differences in diseases and mental disorders. These evolutionary principles are based on evidence obtained from non-human, model organisms such as fruit flies. We acknowledge that extrapolation from a small number of non-human species to humans based on putative evolutionary principles is not necessarily completely reliable, especially with respect to higher-resolution details of mechanism or outcome.

### From Sexual Selection to Male- and Female-Driven Evolution

In *the Descent of Man and Selection in Relation to Sex*, Darwin (1871) noted that as organisms’ sensory functions developed over time, so did their exercise of mate choice during mating and reproduction. Among higher organisms, Darwin (1871) hypothesized that sexual selection was often more important than natural selection. Despite his awareness of males’ often aggressive behavior during the initiation of mating through song, dance, and the pursuit of females, in his theory of sexual selection Darwin mainly focussed on male–male competition and female choice in relation to mate choice. A large body of data has been gathered on male behavior, both generally and during mating, and in light of which it has become clear that *role of sexes in relation to each other* involves more than mate choice (sexual selection) and should be extended to all aspects of mating behavior associated with reproduction (Markow 2002; Singh and Jagadeeshan 2018). For example, males engage in mate search, defending territory, male–male competition, courting, nuptial gifts, mating, mate guarding and sperm competition. Similarly, females engage in mate selection with respect to mate quality, multiple mating, and mate retention. We have introduced the terms *“male-driven evolution”* and “*female-driven evolution*” to capture the extended meaning of sexual selection (Singh and Kulathinal 2005; Jagadeeshan et al. 2015). We are aware that, strictly speaking, some of the behaviors listed here are outside canonical sexual selection; however, our goal is not to redefine sexual selection but to extend it. We need a term that includes all aspects of mate-driven behaviors associated with mating, and we feel that male-driven and female-driven evolution describe the sexes’ roles and impacts on each other’s fitness better than canonical sexual selection (Bateman 1948; Singh and Jagadeeshan 2018). Male–female-driven evolution is especially relevant to human evolution as the sexes have become the most important part of each other’s lifestyle, environment, and ecology. Sexes drive each other.

### Male-Driven Evolution and the “Masculinization” of the Genome

Sex-biased developmental processes are the results of the evolutionary integration of millions of years of small modifications that have accrued differentially in males and females (Singh and Jagadeeshan 2018). For example, although the concept of equal biparental genetic material (autosome) contributions (barring mitochondria and Y) is simple to understand, parental imprinting can be difficult to explain without understanding the theory of sexual antagonism (Arnqvist and Rowe 2005; Bedhomme et al. 2008) and the associated complementation of paternal and maternal genomes involving specific sets of imprinted genes under evolutionary pressure (Kaneko-Ishino et al. 2006). These mechanisms ensure cross-fertilization and the maintenance of genetic variation in populations.

The various sexually dimorphic molecular mechanisms associated with male-biased changes are summarized in Table 1. Starting with the sex-determining gene (SRY), a battery of differently expressed genes is sequentially activated, resulting in sex-based differences in morphology, physiology and behaviors that involve sex-specific, sex-limited, and sometimes non–sex-specific genes. Gene expression studies in *Drosophila* have demonstrated that over 50% of all genes are expressed in a sex-biased manner (Haerty et al. 2007). Males have higher levels of sex-biased gene expression, and male-biased genes display (1) a wider range of gene expression levels, (2) an increased number of male-specific transcripts or unique transcripts and (3) increased rates of molecular evolution resulting from higher DNA mutation rates and/or stronger selection pressures (Haerty et al. 2007; Assis et al. 2012). In *Drosophila* and mice, sperm genes have been shown to evolve faster than other genes with respect to both sequence and coding length.

While males and females may share the same genes, their gene expression patterns (Kang et al. 2011; psychENCODE 2015) and interaction networks (Sousa et al. 2017) can differ significantly due to sex-limited expression (Bedhomme et al. 2008), genomic imprinting (Reik and Walter 2001) and the translocation of genes on the X chromosome (Rice 1994). Parsch and Ellegren (2013) showed sex-biased expression can accelerate the evolution of sex-linked genes. Lemos et al (2010) showed the role of Y chromosome of Drosophila as a major source of epigenetic variation in natural populations that can modulate the expression of biologically relevant phenotypic variation. In humans, Xue et al. (2014) examined sex-biased gene expression in the prefrontal cortex and found the largest fraction of sex-biased genes expressed outside gonadal tissues.

These sex differences likely come from male-driven evolution (Singh and Kulathinal 2005). The male sex drive intensifies male–male competition for sex (Anderson 1994), and only a small percentage of males may be allowed to mate. The male sex drive is *stronger* and *self-enforcing*, inducing the rapid evolution of sex-related fitness traits over time, which can generate cascading pleiotropic effects between the sexes.

### Higher Rates of Male-Biased Mutations and Sexual Dimorphism in Disease and Health

Most estimates of the germline mutation rate show a male bias (Wilson-Sayres and Makova 2011). Often, they are based on the frequency of population-wide autosomal dominant diseases that result from new mutations in one parental gamete and represent the direct observation of mutations in genes and genomes in parents and their offspring. Over the years, these diseases—including endocrine neoplasia type 2A (MEN2A) (Carlson et al. 1994), Apert syndrome (Moloney et al. 1996) and Noonan syndrome (Tartaglia et al. 2010)—have been recognized as showing predominantly paternal origins. Today, a direct estimate of the mutation rate is being generated using gene and genome sequences. The 1000 Genomes Project identified a de novo mutation rate ranging from 1.0 × 10^−8^ to 1.4 × 10^–8^, with an average of µ = 1.1 × 10^–8^ (Genomes Project Consortium 2012). A more recent assessment based on whole-genome sequencing performed in 78 trios from Iceland found a mutation rate of µ = 1.20 × 10^–8^ (Besenbacher et al. 2015). These results imply that, on average, as many as 100 de novo mutations arise in a newborn, with a net fitness loss of about 1%. Many diseases often exhibit a paternal age effect (i.e. an association with the age of the father at the time of the affected child’s birth), and it is expected that a large proportion of de novo mutations come from the father. Male mutation rates increase twofold between the ages of 20 and 40 and at a much higher rate thereafter (Crow 1997). Germline mutations become part of the genetic architecture of the population, and their impacts are realized due to their expression in time and space, which may be sex-specific (Rigby and Kulathinal 2015), particularly among genes expressed in the brain (Trabzuni et al. 2013). Stanley and Kulathinal (2016) showed that neurogenic genes were on average longer in size than non-neurogenic genes in coding regions, untranslated regions, and miRNA and transcription factor binding sites. Longer neurogenic genes would provide a larger mutational target for behavioral change.

These results provide a foundation for the existence of a differential genetic threshold necessary for the manifestation of deleterious mutations between the two sexes. Based on different gene numbers (due to X and Y), expressions and sex-specific thresholds, a given set of disease-causing mutations may be sufficient to manifest a disorder in one sex but not the other. Additionally, a deleterious mutation may manifest mildly and early during development in only one sex. This is likely to be the case for gene mutations associated with essential autism, which represents a milder form of the disorder and is much more frequently identified in males. Conversely, an identical genomic combination present in a female may generate a different physiological threshold due to female-specific expression. The female-specific threshold may thus not be sufficient for the manifestation of the disorder, which would allow females to be more tolerant of and protected against deleterious mutations (Cauvet et al. 2019). More importantly, any manifestations of the disorder in females may require more severe mutations, including major deletions and duplications. These mutations may affect the developmental dysmorphologies common in “complex” autism, which occurs more frequently in females. We suggest that this interactive model is under constant pressure due to differential mutations in the parental germline and the developing brain, sex-specific epigenetic regulatory mechanisms, and the realization of sex-specific metabolomics (the threshold for disease manifestation).

### Male-Driven Evolution and Female Fitness Modification

To the extent that males and females have different lifestyles, they can affect each other’s survival and reproductive success. Male mating behavior has one of the most unexpected and consequential effects on women’s health, namely affecting women’s fertility and longevity. We demonstrate this with respect to the origins of menopause and post-menopausal longevity. There is a tendency for men to prefer to mate with younger women in all cultures and this shows up in the age differences of the couples. United Nations population surveys show that as late as 2014, average age of men at first time marriage is 2–5 years higher than that of women (United Nations Population Facts 2016). Age differences are higher is developing countries which suggests the age difference must have been larger in the past. History shows that through polygamy, serial monogamy, harems and brothels, men have maintained a preference for younger women. From an evolutionary point of view, all else being equal, early reproduction pays off; however, the preference for younger women has produced a major maladaptive effect in women, ultimately resulting in the loss of reproductive ability after menopause. The preference for younger women means depriving older women of the opportunity to reproduce, which could have led to the accumulation of female infertility mutations giving rise to menopause (Morton et al. 2013; Takahashi et al. 2017; Chan et al. 2020). A correlated negative response to this male mating behavior has been the persistence of *maternal mortality* due to repeated cycles of *“younger mates–maternal mortality–serial monogamy–younger mate”* (Jagadeeshan et al. 2019). Male-driven mate choice through older males mating with younger females promotes longevity in both sexes, as older, long-living males contribute their genes to both their sons and daughters (Tuljapurkar et al. 2007), thus solving one of evolutionary biology’s paradoxes, namely why women live beyond menopause (Hamilton 1966). The evolution of menopause is a major factor in women’s health. While male-driven evolution causes a major loss of female fitness via menopause, its effects on female immunity is supposed to be increased via antagonistic evolution.

### Male-Driven and Male-Benefiting Mutations, Antagonistic Evolution, and Female Immunity

While the role of male-driven sexual selection in the evolution of senescence is well known (Williams 1957; Kirkwood and Rose 1991), the role of male-driven, male-benefiting mutations that are harmful to female in promoting female immunity through antagonistic evolution has only relatively recently been investigated and is consequential to women’s health (Arnqvist and Rowe 2005; Bedhomme et al. 2008). The discovery of higher rates of *male-biased mutations* has added to the importance of male-driven antagonistic evolution in females. The combined effects of the evolution of senescence through male–male competition for early fitness gains (Williams 1957; Kirkwood and Rose 1991) and females’ antagonistic responses to protect themselves from the deleterious effects of male-benefiting mutations (Arnqvist and Rowe 2005; Bedhomme et al. 2008) can lead to differential rates of senescence as well as *higher thresholds* for diseases and mental disorders in females (Morrow 2015). This higher level of inherent immunity underlies the generally lower prevalence of diseases in women. In addition, compared to men, women generally exhibit fewer health-affecting conditions as they age.

### Rapid Evolution and the Complexity of the Genome: The Runaway Brain

Mental disorders are generally complex and present with varying symptoms due to several contributing factors. First, the brain is a complex structure, and the various regions of the brain likely overlap in structure and function; therefore, the neurons that affect a given behavior can be found throughout the brain (Sofroniew 2017). Second, brain functions such as communication require the engagement of multiple cognitive centers—such as those responsible for memory, reason, logic, and emotion—and the simultaneous interconnection of multiple brain function centers may result in long-term coordinated evolution and interdependence. Third, as proposed by King and Wilson (1975), the rapid evolution of the brain supposedly takes place through regulatory gene evolution (Chen et al. 2018). Fourth, as is the case for any trait under strong selection pressure and rapid evolution, multi-region brain interactions are likely to be affected by population genetic constraints imposed by factors besides genetic variation, such as genetic linkage, selective sweep, background selection, drift-driven fixation of deleterious mutations, pleiotropy, and the evolution of complex and shared functions (Guan et al. 2019). Finally, because of the wider tissue distribution of brain genes, brain-affecting mutations would be expected to hit a larger target of traits than other mutations. Brain tissue–expressed genes in humans have been shown to be of larger size on average, thus providing a larger *mutational target* (Stanley and Kulathinal 2016). Several mental disorders, such as attention deficit hyperactivity disorder, major depressive disorder, schizophrenia, and bipolar disorder, have been shown to be affected by common neurotransmitters, including serotonin, dopamine, norepinephrine, and glutamate systems (CDGC: Cross-Disorder Group of the Psychiatric Genomics Consortium 2013). Consequently, a single neurotransmitter may have multiple effects associated with various mental disorders.

Neurodevelopment is complicated and involves several unusual features. Unlike other organs, the brain is characterized by long periods of development, differentiation, pruning and maturity. Neurodevelopment starts early in organismal development and can continue for decades in humans. This developmental continuum is highly sensitive to environmental effects, and even single exposures to adverse environments can leave lifelong imprints. For example, prenatal alcohol exposure is the primary cause of fetal alcohol spectrum disorder (FASD), a common developmental disorder characterized by lifelong behavioral abnormalities, including learning and memory deficits (Chokroborty-Hoque et al. 2014). Also, neurodevelopment involves unusual postzygotic somatic mutations which may involve a variety of mutational mechanisms, including transpositions (Krupp et al. 2017; Lim et al. 2017) and rare copy number variants (Wilfert et al. 2017). These mutations appear de novo during ontogeny, play an important role in neuronal differentiation and result in every brain’s representing a genetically unique mosaic. Postzygotic mutations constitute a significant proportion of de novo mutations and may contribute to the liability threshold of developing brain disorders (Singh, Castellani and Hill 2020). How the contributions of individual-specific de novo mutational events contribute to neurodevelopment remain unclear, as does whether these events occur similarly or differently in male and female brains. If sex-specific differences in somatic mutations occur, as with germline mutations, these mutational events may contribute to the increased incidence of autism in males, adding yet another source of sex-related differences that can affect individual brain function. The increased mutation rate observed in males compared to females may cause higher genomic heterogeneity through spermatogenesis, which is transmitted to both sexes and expressed differently in each, as well as somatic de novo mutations during neurodevelopment, which may preferentially affect males. In both cases, these mutations are sources of novel variations and the generation of extreme phenotypes associated with a unique gamete and a unique mosaic brain.

A recent study (Shi et al. 2016) performed a time series analysis of genome-wide transcription profiles from human brains at major developmental stages (prenatal, early childhood, puberty and adulthood) and showed that more than 2000 genes exhibited between-sex divergences during major developmental stages, with the greatest number found at puberty (4164 genes). More importantly, the male-biased genes were found to be highly enriched *“for genes involved in neurological and psychiatric disorders like schizophrenia, bipolar disorder, and autism, while no such pattern was seen for the female-biased genes”* (Shi et al. 2016). Such results directly support the theory that male-driven mutations and gene expression have the potential to represent a major source of sex bias in some mental disorders.

The rapid evolution of the brain increases the likelihood that brain-expressed genes display shared functions and pleiotropic effects. Some evidence supports male-biased increases in physical growth (de Zegher et al. 1999) with respect to birth length, weight and head circumference (Reuter et al. 2018), which may cause male-biased birth complications and could potentially affect neuronal development. A faster fetal growth rate may also increase vulnerability to developmental perturbations with pleiotropic effects on mental capacity genes and behavioral faculties in a sex-biased manner.

## The Theory

**Fig. 1:**
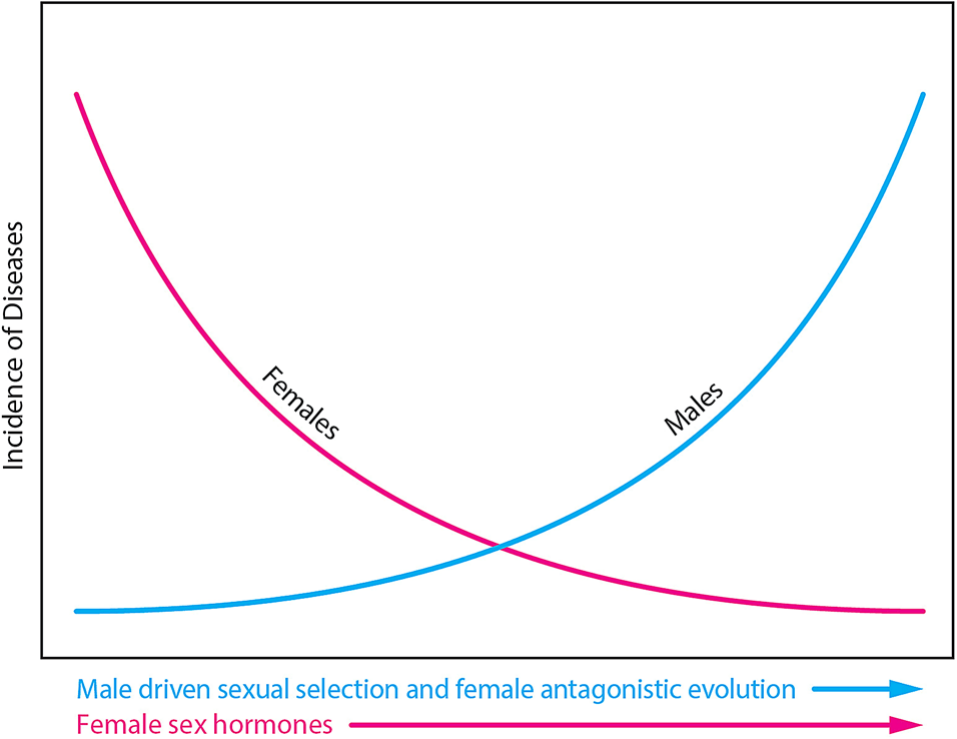
Role of male-driven sexual selection and female sexual hormones in the incidence of sex-biased diseases and mental disorders under the *Sexual Selection-Sex Hormone* Theory (see text for explanation)

Sexual dimorphism is prevalent across mammalian species (Karp et al. 2017) and it occurs in many human complex traits including diseases and mental disorders (Rawlik et al. 2016). Barring the effects of lifestyle and work-related causes, sex bias in diseases and mental disorders can be attributed to two main biological causes: male-driven evolution and female sex hormones (Fig. 1). The former is the result of male–male competition for fitness gains and the evolution of male-benefiting and female-harmful mutations leading to the antagonistic evolution of female protection. A hypothetical example of male-benefiting mutation or trait that is harmful to females can go like this: Male sexual desire during pregnancy would be risky to the fetus, and it would be better for women to evolve low sexual desire and/or higher rejection during pregnancy. Sex hormones have been shown to provide female protection (Abel et al. 2010). This is sensible from an evolutionary perspective, as females are more important than males for population fitness. Sex hormone fluctuation during reproductive years has been shown to increase female vulnerability to diseases such as migraine (Artero-Morales et al. 2018) and depression (Brummelte and Galea 2016; Slavich and Sacher 2019) and is highly sensitive to environmental and social conditions. As a result, it can be expected to vary across communities and countries (Slavich and Sacher 2019). Health is complicated; but other factors must be involved. Sexual Selection—Sex Hormone hypothesis predicts that due to the combination of the two factors, females will show greater resistance to physiological disruption or dysfunction due to mutation or epimutation. Furthermore, the theory predicts that female-biased ratios be higher for sex hormone-affected diseases and disorders.

**Fig. 2:**
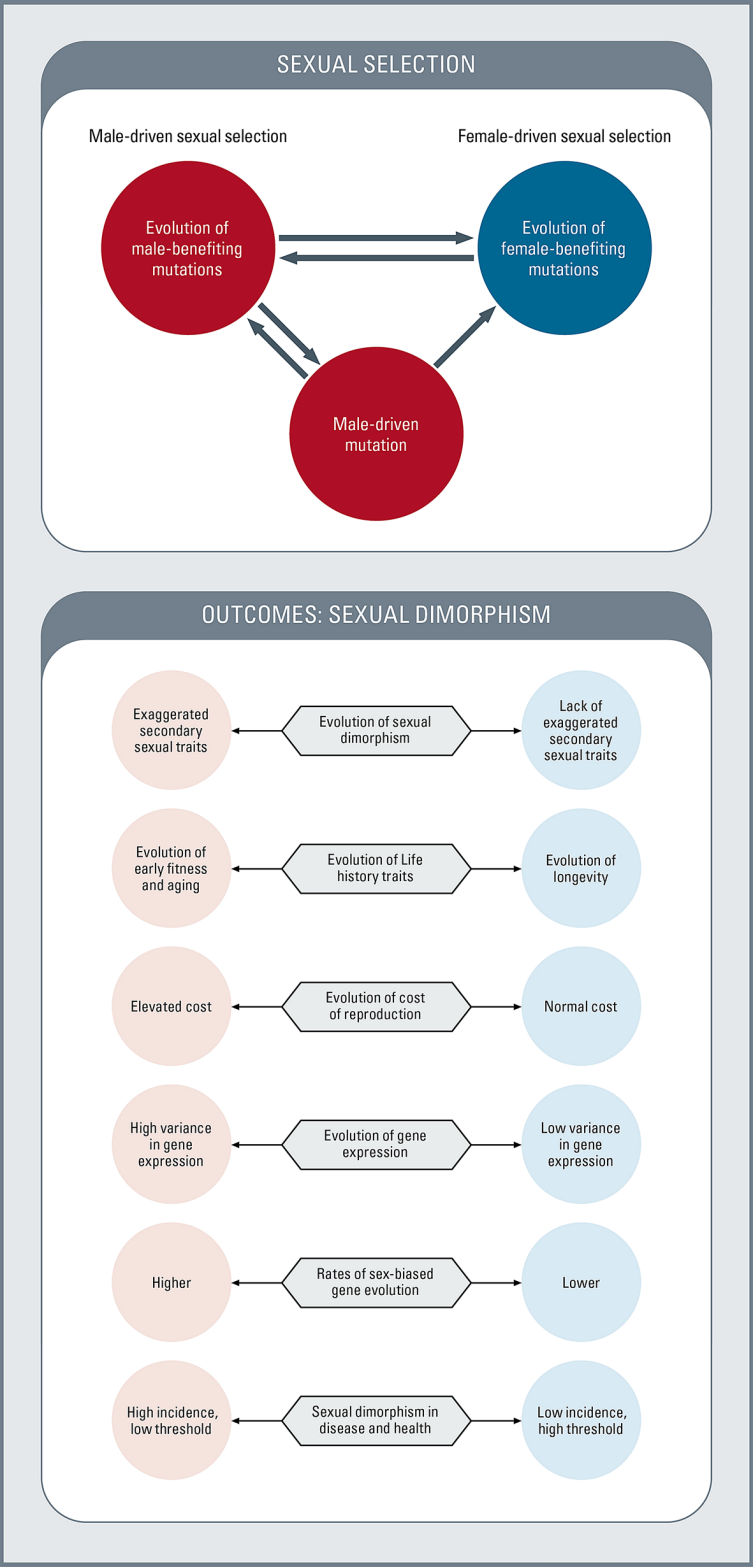
Male-driven and Female-driven sexual selection and their effects on sexual dimorphisms in morphology, physiology, gene expression, and life history traits

**Fig. 3:**
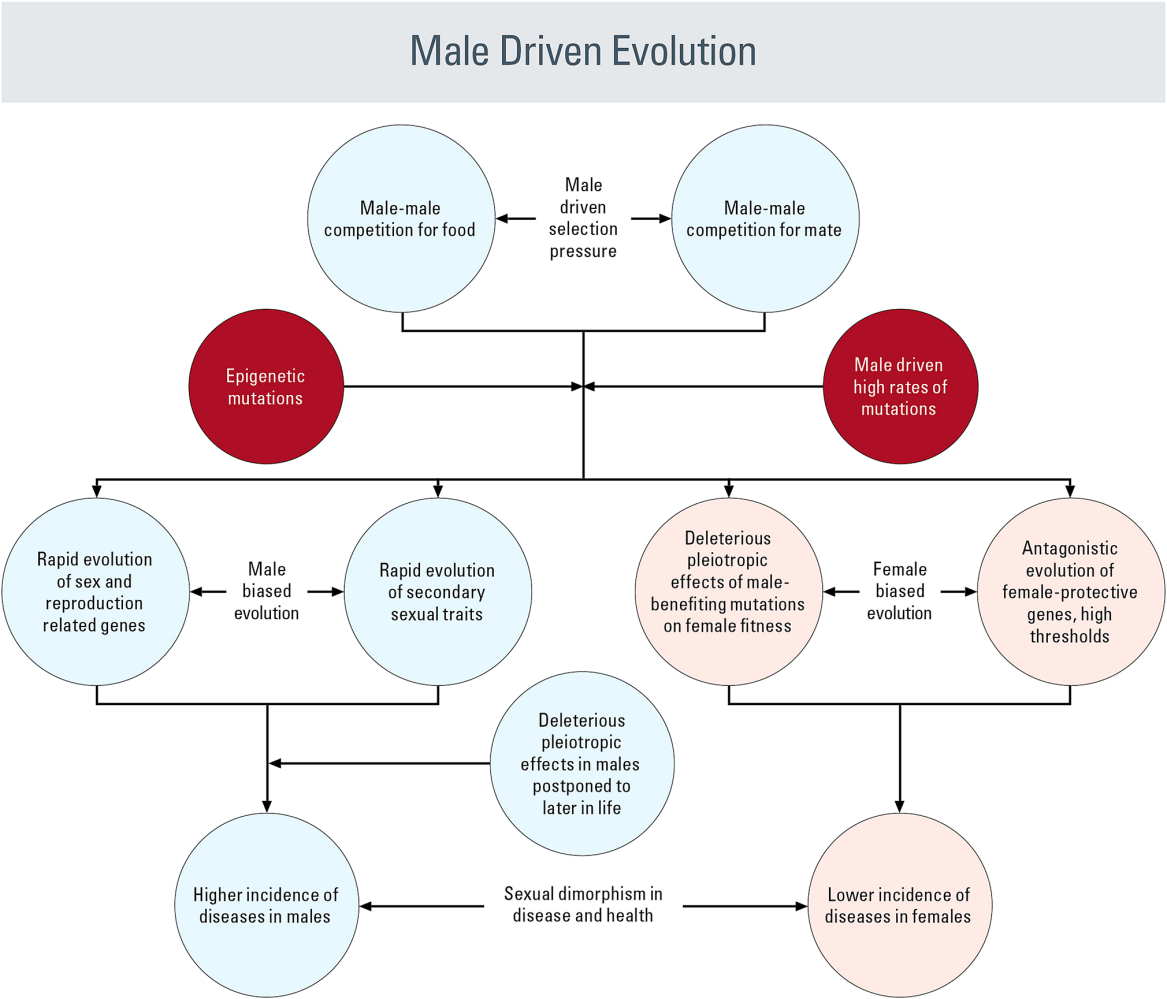
Schematic representation of male-driven and female-driven evolutionary mechanisms, sexual antagonism, and sexual dimorphisms in disease and health

The processes and outcomes of male-driven evolution are depicted in Fig. 2, while Fig. 3 shows how it leads to the evolution of male vulnerability and female immunity. Male-driven evolution–sex hormone theory posits the following: (1) due to the stronger male sex drive, male–male competition for mates and the opportunity for repeated mating in continuously breeding populations, male-driven selection pressure is stronger and perpetual; (2) male-driven selection pressure, in combination with higher rates of male-driven mutation, has powerful effects on evolutionary changes in both general and male-biased traits; (3) rapid sexual selection–driven changes in combination with male lifestyles favoring early fitness gains foster the accumulation of late-acting deleterious mutations, with pleiotropic health effects and a general loss of fitness in later life; and (4) sexual selection–driven changes in gene expression that are beneficial to males but harmful to females place additional pressure on females to respond through antagonistic evolution, giving rise to higher female immunity and higher thresholds for exhibiting disease symptoms (Arnqvist and Rowe 2005).

**Fig. 4:**
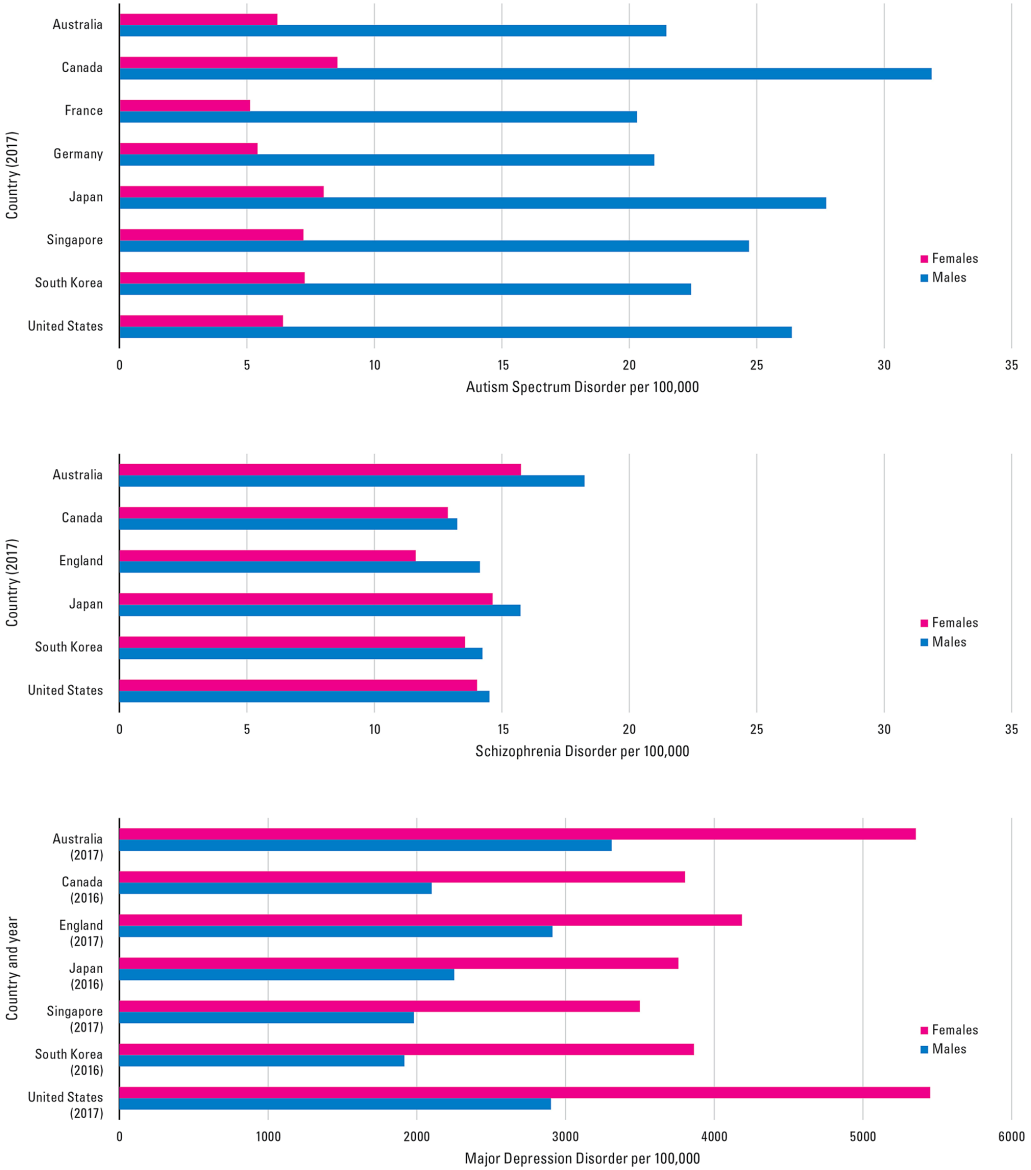
Estimated age-standardized incidence rates of autism, schizophrenia, and major depression in both sexes, all ages

The combined effect of the Sexual Selection–Sex Hormone theory is demonstrated in the three examples shown in Fig. 4. This graph is meant to serve as a general illustration. The three examples of mental disorders were chosen to show the effect of male-driven sexual selection in the case of autism, sex hormone in the case of major depression disorder, and possibly both in the case of schizophrenia. The hypothesis presented here can explain the sex-biased patterns of these disorders but remains to be experimentally investigated. The lifetime incidence of schizophrenia in men and women is not very different; the incidence is higher among men aged 15–30 and moderately higher in women aged 45–60 (Abel et al. 2010). It is likely to be more complicated (Li et al. 2017) but the underlying causes of schizophrenia may be *sex-specific modulators*, as women have been shown to suffer less than men from cognitive deficits (Goldstein et al. 1998).

The reduced sex disparity in de novo mutation-cased autism may be the result of evolution. According to the hypothesis outlined above, sex bias is the result of evolution, not simply of mutation & variation. Male-driven germline variants get inherited by both sexes but have not had the time to go through sex-biased evolutionary modifications. Unlike segregating common variants associated with essential autism, rare de novo mutations of large effect (associated with complex autism) may face sperm competition including lethality in early embryonic and/or fetal development. We may add that abortuses are known to carry increasing number of large effect mutations and are sex biased (higher for males) as there is no data to reliably reflect on their direct involvement in autism.

## Supporting Evidence

Male-driven evolution and sexually antagonistic evolution are major forces of sexual divergence (Figs. 2 and 3). Almost everything we know about sex-biased diseases can be explained by this evolutionary hypothesis. It is, however, important to point out that since the hypothesis being presented here straddles between two different disciplines- evolutionary biology and Medical sciences, stronger test of the hypothesis will come from new testable predictions and new observations. In the following subsections, we summarize available evidence and make predictions that would support the hypothesis.

### Male Bias in Autism is Greater for “Essential” and Less Severe Autism Cases

Several key observations regarding the nature of sex differences in autism support the male drive theory (Chakrabarti and Fombonne 2001; Miles 2011). This theory predicts that a significant proportion of autism cases may be caused by mutations in large numbers of non-pathological genes, which appears to be the case (Constantino and Todd 2000). The theory also predicts that the genes involved will likely be longer, interact with other genes and be associated with extensive pleiotropic effects. Furthermore, these genes may differ in number and be differentially expressed in the two sexes, with the potential to offer a lower threshold for the highly variable manifestation of the essential (less severe) form of autism in males.

### Females Show Fewer Cases of Diagnosed Autism but More Severe Symptoms

This is expected based on the theory of antagonistic evolution. If the severe male bias observed for essential (less severe) autism is due to the pleiotropic effects of male-driven minor or multifactorial mutations, females would be expected to be selected for protection against these mutations through sexually antagonistic evolution. Conversely, major mutations—de novo or otherwise—would affect both sexes equally. The lower incidence of autism in girls may result from female-favoring protection mechanisms due to sexual antagonism effects (Rice and Chippindale 2001; Bedhomme et al. 2008). Male bias appears to be more pronounced for less severe forms of autism, which would be expected according to the theory of mutation–selection balance. We would expect that milder autism, which results from gene mutations that segregate in the population, would be moderated by antagonistic effects in females and exhibit greater male bias than severe autism. Women differ from men in immune response and show higher levels of circulating antibodies and greater cytokine production in response to infection. The higher immunity of females as compared to males is phylogenetically conserved suggesting adaptive advantage in reproductive success (Klein and Flanagan 2016; Fink and Klein 2018).

### The Effects of Male-Biased vs. Female-Biased Disease-Causing Genes and Alleles are Expected to be Negatively Correlated

This follows directly from antagonistic selection theory. If male-biased diseases are the pleiotropic effect of male-benefiting mutations, such mutations will be suppressed in females, giving rise to allele-specific immunity. In a recent study, Kamitaki et al. (2020) demonstrated the role of sex differences in complement protein levels in producing sexual dimorphism in diseases, i.e., a negative correlation between the effects of C4 alleles in men and women (on schizophrenia in men and on systemic lupus erythematosus [SLE] and Sjogren’s syndrome in women). This appears to fit the models of intra-locus sexual conflict resolution (Bedhomme et al. 2008). While we expect that more such genes will be found, sexually dimorphic disease genes need not all be allelic; the majority arise from intergenic resolutions of sexual conflicts (Parker and Patridge 1998).

### Epigenetic Responses to Environmental Challenges are more Likely to Occur in Males than in Females

Recent developments in the field of epigenetics have added yet another mechanism that may determine phenotypic differences and be subject to natural selection. Increasing evidence suggests that some of these features can be transmitted to the next generation and affect evolutionary changes (Bjornsson et al. 2004; Nadeau 2009). Evolution via epigenetic changes is not well understood but may include alterations in DNA methylation, which is directly associated with gene expression patterns and known to be responsive to environmental exposures. DNA methylation is also known to differ between the sexes and can account for sexual dimorphism (Martin et al. 2017) and the male-biased frequencies of disorders. Given the extensive involvement of environmental effects in the development and functioning of the brain, DNA methylation likely plays a role in the development of mental disorders (Castellani et al. 2015).

A recent study of sex-specific DNA methylation rates with a cohort of 72 participants who had experienced environmental chemical exposure (Leung et al. 2018) reported that increased chemical exposure resulted in more frequent DNA methylation changes in males (4 in 16) compared to females (1 in 16), although this difference was not significant. Moreover, the rate of DNA methylation in males was over a thousand times the rate in females. The study found that 15% of male-specific CpG sites were enriched in the cytobands of the X chromosome that have been associated with neurological disorders. The researchers argued that sexually dimorphic responses to environmental exposures may contribute to the increased frequencies of some disorders, including brain disorders, in males compared to females.

### De novo Neurodevelopmental Mutations Involved in the Mosaic Brain may Occur more Frequently in Males than Females

The genome varies not only across individuals but also from cell to cell, making every individual a unique mosaic (Freed et al. 2014). Although mosaicism is not uncommon in mammals, it occurs much more extensively in the mammalian brain than in other organs. Mosaicism occurs due to de novo neurodevelopmental somatic mutations, which result in every human brain’s representing a unique combination of genomic variations. The impact of this phenomenon is directly realized in the resulting individual. Mutation rates have been shown to vary widely in somatic tissues, from 8 times in the brain to 122 times in skin cells (Tomasetti et al. 2013; Lodato et al. 2015; Martincorena et al. 2015; Lynch 2016) with an average of 50-fold inflation. This means that a somatic cell will contain approximately 5000 (100 × 50) de novo mutations. Even the eightfold inflation in the brain will produce 800 new mutations per cell. Since all cells within a tissue will not have the same set of mutations, many genes within a tissue will have been mutated, and this number will be larger in the male brain than in the female brain. Some of these mutations may cause and contribute to a variety of neurodevelopmental disorders, including autism, that have been linked to de novo mutations (Ronemus et al. 2014; Richter et al. 2019; Uddin et al. 2018). Additionally, these mutations can affect even healthy individuals, causing variable manifestations including variable thresholds for responses to external exposures.

### Clinical Significance

One might ask whether clinicians and physicians need to know about evolution. The answer is yes. The cells in our bodies have a long evolutionary memory, and if clinicians are to understand the biology of a disease, they must also be aware of evolutionary principles in general and how they impact health related to sex and gender. Physicians often ask patients about their parents and siblings, whether they are living and, if not, how they died. This information provides the physician with a general biological prognosis of a family’s health. A surgeon at the operating table may need to be aware of varying dose responses to drugs such as anesthesia and blood thinners in different ethnic populations. The number of situations that involve relevant genetic contributions will continue to increase with advances in molecular insights and precision medication. Cancer clinicians are becoming increasingly interested in understanding mutation–selection clonal dynamics associated with tumor growth, which may facilitate individualized medicine. The medical establishment is becoming increasingly aware of the importance of genetic differences and of sex and gender when assessing health and disease. Therefore, all health researchers must be aware of population diversity, unique individuality, drug complexity and the possibility of gene–drug interactions. As we continue to learn about the genome and move towards precision medicine, physicians and clinicians will need to know about not only the contributions of genetics but also the evolutionary history of these changes. H. J. Muller (1950) best expressed the importance of understanding the contributions of genetics and evolution to human health in the context of high rates of mutations and human fitness, although his comments are equally applicable to sex-biased diseases and mental health:

Only after the opposition [of the groups with vested interest] has become sufficiently weakened to allow the conception of evolution, including that of its genetic mechanism, to become as much a cornerstone of elementary education as the rotundity of the earth, and after the processes and consequences of genetic change throughout the ages have been vividly visualized and dramatized for people in general from their early years on through their later development, can we expect the arguments, calculations and recommendations of geneticists to take on sufficiently concrete meaning for the average man, the medical man, and the man in public life, so as to influence them adequately in their conduct of practical matters. To work for this modernization of educational policy and methods, with a view to reshaping the average man’s view of his place in nature, is, therefore, one of the first duties of those who appreciate the significance of genetics in human affairs.

While much has changed in genetics since Muller made those remarks, his remarks still stand with regard to the role of evolution in human health and disease. Origins and patterns of Mendelian diseases have benefited from the principles of population genetics evolution, and many genetic details are being filled in by recent advances in genomic technologies, including single-cell genomics (Macaulay and Voet 2014). We are still far from harnessing the insights of evolution in the origin of sex-biased diseases and precision medicine (Singh and Gupta 2020). Significant sex differences are becoming the focus of attention for many complex diseases. A sex-specific focus on male and male-driven evolutionary causes offers a novel approach to understanding sex-biased complex diseases. Specifically, we argue that autism research would benefit from sex-specific evolutionary approaches.

## Conclusions

With notable exceptions, such as the “thrifty gene” hypothesis of diabetes (Neel 1962) and the evolution of the complex immune system in response to pathogens, the effects of evolution on human health have been relatively ignored, partly due to the uniqueness of each individual and partly due to the immediacy of the problems facing the clinician. In medicine, as in all functional biology, the *why* is often more important than the *how*; however, the field has slowly begun to appreciate that the body has a history and, as the physicist Max Delbruck (1949) remarked, *“any living cell carries with it the experiences of a billion years of experimentation by its ancestors.*”

Although the molecular mechanisms that underlie mental disorders and/or conditions such as autism may be associated with individual risk factors or developmental anomalies, sex-mediated differences in disease prevalence may be associated with the evolutionary history of the sexes. Role of evolution in sex differences is increasingly being appreciated (Morrow 2015; Klein and Flanagan 2016, Fink and Klein 2018). We provide an overarching Sexual Selection–Sex Hormone theory to explain male-biased prevalence of diseases and mental disorders, and higher female immunity. We propose that the male-biased prevalence of mental disorders such as autism may be caused by the joint effects of three processes: (1) *increased male vulnerability* due to the negative pleiotropic effects of male-driven sexual selection and evolution, causing sex-specific differences in gene expression during neurodevelopment; (2) *increased rates of male-driven mutations*, both germline and somatic, due to male-driven sexual selection and resulting early fitness gains at the cost of later fitness; and (3) *increased female immunity* due to the development of antagonistic responses to mutations that are beneficial to males but harmful to females, reducing female vulnerability and increasing the thresholds for the development of mental disorders, including autism. The male-driven processes associated with the rapid evolution of the brain may also result in increased neurodevelopmental male susceptibility. Such insights may help identify differential treatment protocols, including the sex-specific dose–response efficacy of drugs. Researchers and clinicians exploring sexual dimorphism associated with diseases and mental disorders should also consider the health consequences of sexual selection and evolution (Mayr 1961).

## Abbreviations

CDGC: Cross-Disorder Group of the Psychiatric Genomics Consortium
FASD: Fetal Alcohol Spectrum Disorder
